# Genomic and Transcriptomic Insights into Carbon-Source and Temporal Induction of a Diverse Set of Lignocellulolytic Enzymes in *Irpex lacteus* QJ

**DOI:** 10.3390/jof11120882

**Published:** 2025-12-13

**Authors:** Liye Song, Baorui Liu, Qijun Zhu, Kun Meng, Hongying Cai, Yunsheng Han, Weiwei Liu, Peilong Yang

**Affiliations:** Key Laboratory of Feed Biotechnology of Ministry of Agriculture and Rural Affairs, Institute of Feed Research, Chinese Academy of Agricultural Sciences, Beijing 100081, China; songliye_sly@163.com (L.S.); 15853195136@163.com (B.L.); zqj13881197414@163.com (Q.Z.); mengkun@caas.cn (K.M.); caihongying@caas.cn (H.C.); hanyunsheng@caas.cn (Y.H.)

**Keywords:** *Irpex lacteus*, lignocellulose degradation, genome sequencing, transcriptome analysis, cellobiose dehydrogenase, wheat straw

## Abstract

The white-rot fungus *Irpex lacteus* is recognized for its strong ligninolytic and polysaccharide-degrading capacity, but the key advantages in degrading lignocellulose and the regulation of its enzyme systems remain poorly understood. In this study, we identified a rich repertoire of carbohydrate-active enzymes in the genome of *I. lacteus* QJ. Relative to other white-rot fungi, an expanded glycoside hydrolase gene family in *I. lacteus* QJ suggesting strong potential for lignocellulose degradation. When *I. lacteus* QJ was cultivated on glucose or wheat straw for 4 and 8 days, wheat straw strongly induced carbohydrate-active enzyme genes on day 4, while ligninolytic enzyme genes exhibited delayed upregulation on day 8. The cellobiose dehydrogenase plays an important role in the degradation processes. Its expression pattern is consistent with that of cellulase, and it can support peroxidase activity by providing H_2_O_2_. These findings reveal temporal coordination between polysaccharide- and lignin-degrading enzymes, providing new theoretical ideas for the application of *I. lacteus* during the degradation process. Our results not only improve the mechanistic understanding of fungal lignocellulose deconstruction but also inform strategies to enhance biological pretreatment of agricultural residues for biorefinery applications.

## 1. Introduction

Agricultural residues such as straw represent low-cost biomass resources with substantial recycling potential [1]. They can be repurposed in composting, animal feed, biofuel production, and novel material development [1,2,3,4,5]. The primary constituent of straw is lignocellulose, a complex matrix composed of cellulose, hemicellulose, and lignin. The proportions of these three components vary by material source. Wheat straw typically comprises 32–45% cellulose, 20–45% hemicellulose, and 11–26% lignin. In contrast, corn stalk contains 36.89% cellulose, 20.42% hemicellulose, and 17.38% lignin. For straw, cellulose and hemicellulose together represent about 50–80% of the dry matter, whereas lignin contributes approximately 10–15% [2]. Minor amounts of minerals or other constituents may also be present [3].

Cellulose is a linear polysaccharide consisting of glucose units connected via β-1,4-glycosidic bonds; its chains are linked through hydrogen bonds, forming crystalline regions that are insoluble [6]. These crystalline regions hinder enzyme accessibility. Hemicellulose is an amorphous heteropolysaccharide composed of various monosaccharides; it noncovalently connects cellulose and lignin [7]. Xylan is the most abundant polysaccharide within hemicellulose [8,9]. In contrast, lignin is not a carbohydrate but an irregular polymer of aromatic compounds [7]. It is mainly composed of three monolignols: syringyl (S), guaiacyl (G), and p-hydroxyphenyl (H) [10]. The most abundant interunit linkage is the β-O-4 (β-aryl ether) bond, constituting approximately 43–65% of lignin bonds [11]. Within lignocellulose, the tight network formed by the three components hinders degradation. Consequently, the enzymatic hydrolysis of lignocellulose requires the coordinated action of cellulases, hemicellulases and ligninolytic enzymes. The rich polysaccharide content makes lignocellulose an attractive feedstock for fermentation and biofuel production. In industrial processes, pretreatment is often necessary to separate these components and alter the complex structure, thereby promoting enzymatic hydrolysis and subsequent reactions to obtain higher yields of monosaccharides or oligosaccharides [12]. Among published pretreatment methods, biological pretreatments using microbes offer advantages: they do not rely on harsh chemicals, consume less energy, and are more cost-effective and environmentally friendly than physical or chemical methods, making them scientifically valuable [12,13].

In natural ecosystems, filamentous fungi exhibit diverse lifestyles and host ranges, which drive their evolutionary adaptability and endow them with remarkable biocatalytic potential [14]. They secrete robust lignocellulolytic enzymes with the ability to degrade plant residues, positioning them as key players in lignocellulose decomposition [15]. Among these fungi, white-rot fungi are particularly significant because of their ability to fully degrade lignin into CO_2_ and H_2_O [16]. White-rot species produce not only ligninolytic enzymes but also cellulases and a suite of hemicellulases providing high potential for biorefinery and biomass conversion applications [17]. *Irpex lacteus*, a polypore fungus within the white-rot group, has a history of use in traditional Chinese medicine for treating hypertension, edema, and oliguria; its polysaccharides have been developed into a therapeutic agent (“Yishenkang”) for chronic glomerulonephritis [18]. However, in recent years, its capacity for lignocellulose decomposition has garnered increasing attention due to its effective promotion of wood decay. Studies have shown that *I. lacteus* can process wheat straw and wheat straw fermentation by *I. lacteus* results in significantly reduced lignin content compared to *Phanerochaete chrysosporium* and *Pleurotus ostreatus*, demonstrating its selective lignin degradation capability [19].

In the microbial degradation of lignocellulose, the key enzymes include cellulases, hemicellulases, and ligninolytic enzymes [2]. The cellulase system comprises endoglucanases (EGLs), cellobiohydrolases (CBHs, also known as exoglucanases), β-glucosidases (BGLs), and auxiliary enzymes (AAs) [15]. Hemicellulose degradation primarily involves endo-β-1,4-xylanases and β-xylosidases. However, due to the structural complexity of hemicellulose, additional enzymes such as α-L-arabinofuranosidases, α-1,2-glucuronidases, acetylxylan esterases, β-xylobiohydrolases, and esterases also contribute. The major ligninolytic enzymes include laccase, lignin peroxidases (LiP), manganese peroxidases (MnP), versatile peroxidases (VP), and dye-decolorizing peroxidases (DyP) [9,20]. It is essential to conduct a thorough investigation of its degradation mechanisms, including the enzyme types involved and their temporal expression patterns, given the complexity of the lignocellulolytic enzyme system.

Carbon source type notably influences the gene expression of cellulases and hemicellulases in filamentous fungi [21,22,23]. It has been observed that in *I. lacteus*, expression of cellulase and hemicellulase genes is repressed in the presence of simple carbon sources [24]. However, the regulatory mechanisms controlling ligninolytic enzyme expression remain unclear. Additionally, few studies have linked the expression of ligninolytic enzymes with that of cellulases and hemicellulases, and the influence of cultivation duration on enzyme gene expression remains underexplored. In summary, the conditions and regulatory mechanisms that drive lignocellulolytic enzyme gene expression in *I. lacteus* are not fully understood. Further research on the types and mechanisms of enzymes secreted by *I. lacteus* is needed to broaden its potential applications in lignocellulose degradation [25,26].

Our laboratory isolated a filamentous fungus strain with lignocellulose-degrading capability from wheat straw and designated it as QJ. Phylogenetic analysis based on ITS-rDNA identified it as *I. lacteus*. Qualitative assays using guaiacol agar plates demonstrated its strong ligninolytic enzyme activity. In this study, we obtained a draft genome sequence of *I. lacteus* QJ and performed genome annotation to evaluate its potential for biomass degradation. We systematically annotated its lignocellulolytic enzyme genes, including cellulase, hemicellulase, and ligninolytic enzyme families, to characterize its synergistic degradation network by identifying the types and numbers of relevant genes. Subsequently, we cultivated the fungus using wheat straw or glucose as carbon sources and harvested fungal mycelia on day 4 and day 8 from each culture. Transcriptome sequencing (RNA-seq) was performed on the collected mycelia. Differential expression genes (DEGs) analyses revealed how the lignocellulolytic enzymes were regulated in response to different carbon sources and sampling times in *I. lacteus*. Moreover, transcriptome profiling provided insight into the gene expression levels involved in substrate degradation, elucidating underlying biological mechanisms. Collectively, these findings enhance our understanding of *I. lacteus*-mediated lignocellulose degradation with wheat straw as a carbon source and support more effective utilization of agricultural residues.

## 2. Materials and Methods

### 2.1. Strains and Substrate

The fungal strain *I. lacteus* QJ was isolated from naturally fermented wheat straw and preserved at China General Microbiological Culture Collection Center (accession number CGMCC No. 41264).

The media used in this study included: (1) potato dextrose broth (PDB; Solarbio, Beijing, China); (2) potato dextrose agar (PDA; Solarbio, Beijing, China); (3) MA medium: Na_2_HPO_4_·12H_2_O 17.907 g/L, (NH_4_)_2_SO_4_ 1.4 g/L, KH_2_PO_4_ 2.0 g/L, MgSO_4_·7H_2_O 0.60 g/L, CaCl_2_ 0.60 g/L, urea 0.3 g/L, Tween 80 0.5 mg/mL, peptone 2.0 g/L, and MA trace element solution at 1.0 mg/mL. The trace element solution comprised FeSO_4_·7H_2_O 5.0 g/L, MnSO_4_·H_2_O 1.6 g/L, ZnSO_4_·7H_2_O 1.4 g/L, and CoCl_2_·2H_2_O 2.0 g/L; (4) MA-Glu or MA-WS medium, which contained glucose or wheat straw (10 g/L) as the carbon source in MA medium [27].

### 2.2. Sample Culture

*I. lacteus* QJ was first cultured on PDA for five days at 30 °C to obtain fresh mycelium. A 10 mm diameter agar plug from the colony’s edge was then inoculated into 100 mL of PDB for submerged culture. Three replicates were incubated at 30 °C and 200 rpm for four days. Cultures were cooled to 4 °C, centrifuged at 4000 rpm for 10 min, and the pellet was washed with sterile water for genome sequencing. To prepare samples for RNA sequencing, 10 mm agar plugs were inoculated into 100 mL of MA-Glu or MA-WS media (one plug per flask), with incubation at 30 °C and 200 rpm. Each medium type was subdivided into two groups, cultured for 4 days and 8 days, respectively. Then cultures were cooled to 4 °C, centrifuged at 10,000 rpm for 10 min, and the resulting pellets were collected for transcriptomic analysis.

### 2.3. Genomic Sequencing and De Novo Assembly

Genomic DNA was extracted with the CTAB [28]. The harvested DNA was detected by the agarose gel electrophoresis and quantified by Qubit^®^ 2.0 Fluorometer (Thermo Scientific, Waltham, MA, USA). After DNA extraction, library construction was performed. Libraries for single-molecule real-time (SMRT) sequencing was constructed with an insert size of 20 kb using the SMRT bell TM Template kit, version 1.0. At last, the library quality was assessed on the Qubit^®^ 2.0 Fluorometer (Thermo Scientific) and detected the insert fragment size by Agilent 2100 (Agilent Technologies, Santa Clara, CA, USA). A total amount of 1 μg DNA per sample was used as input material for the DNA sample preparations. Sequencing libraries were generated using NEBNext^®^ Ultra™ DNA Library Prep Kit for Illumina (NEB, Ipswich, MA, USA) following manufacturer’s recommendations. At last, PCR products were purified (AMPure XP system) and libraries were analysed for size distribution by Agilent2100 Bioanalyzer and quantified using real-time PCR. The whole genome of QJ was sequenced using PacBio Sequel platform and Illumina NovaSeq PE150 at the Beijing Novogene Bioinformatics Technology Co., Ltd. (Beijing, China). First, perform preliminary assembly with SMRT Link v5.0.1 of QJ. In order to ensure the accuracy of the subsequent analysis results, the low-quality reads were filtered (less than 500 bp) to obtain Clean data. Using the automatic error correction function of SMRT portal, the long reads were selected (more than 6000 bp) as the seed sequence, and the other shorter reads were aligned to the seed sequence by Blasr, so that the accuracy of the seed sequence could be improved further. After assembling we obtained an initial result. Then correct the results of the preliminary assembly. By the variant Caller module of the SMRT Link software, the arrow algorithm was used to correct and count the variant sites in the preliminary assembly results.

### 2.4. Genome Component Prediction and Gene Function Annotation

Genome component prediction included the prediction of the coding gene, repetitive sequences and non-coding RNA. The steps were proceeded as follows: (A) ab initio gene finding using a selection of the following software tools: GeneMarkHMM (https://genemark.bme.gatech.edu/genemark/gmhmme.cgi, accessed on 20 January 2025), FGENESH (https://www.softberry.com/berry.phtml?topic=fgenesh&group=programs&subgroup=gfind, accessed on 20 January 2025), Augustus (http://bioinf.uni-greifswald.de/webaugustus/prediction/create, accessed on 20 January 2025), and SNAP (version 6.0), GlimmerHMM (https://ccb.jhu.edu/software/glimmerhmm/, accessed on 20 January 2025); (B) protein homology detection and intron resolution using the GeneWise software (https://www.ebi.ac.uk/jdispatcher/psa/genewise, accessed on 20 January 2025) and the uniref90 non-redundant protein database; (C) alignment of known ESTs, full-length cDNAs, and most recently, Trinity RNA-Seq assemblies to the genome; (D) PASA alignment assemblies based on overlapping transcript alignments from step (C); (E) use of EVidenceModeler (EVM) to compute weighted consensus gene structure annotations based on the above (A, B, C, D); (F) use of PASA to update the EVM consensus predictions, adding UTR annotations and models for alternatively spliced isoforms (leveraging D and E).

The interspersed repetitive sequences were predicted using the RepeatMasker (http://www.repeatmasker.org/, accessed on 20 January 2025). The tandem Repeats were analyzed by the TRF (Tandem repeats finder). Transfer RNA (tRNA) genes were predicted by the tRNAscan-SE. Ribosome RNA (rRNA) genes were analyzed by the rRNAmmer. sRNA, snRNA and miRNA were predicted by BLAST (https://blast.ncbi.nlm.nih.gov/Blast.cgi, accessed on 20 January 2025) against the Rfam database (https://rfam.org/, accessed on 20 January 2025). We used seven databases to predict gene functions. They were Gene Ontology (GO), Kyoto Encyclopedia of Genes and Genomes (KEGG), COG (Clusters of Orthologous Groups), Non-Redundant Protein Database databases (NR), Transporter Classification Database (TCDB), P450, and, Swiss-Prot. A whole genome Blast search (E-value less than 1 × 10^−5^, minimal alignment length percentage larger than 40%) was performed against above seven databases. The secretory proteins were predicted by the Signal P database (https://services.healthtech.dtu.dk/services/SignalP-6.0/, accessed on 20 January 2025).

### 2.5. RNA Extraction, Library Construction, and Sequencing

Total RNA was extracted from the mycelia using TRIzol^®^ Reagent (Thermo Fisher, MA, USA) according to the manufacturer’s instructions. Then RNA quality was determined by 5300 Bioanalyser (Agilent) and quantified using the ND-2000 (NanoDrop Technologies, Wilmington, DE, USA). Only high-quality RNA sample (OD260/280 = 1.8~2.2, OD260/230 ≥ 2.0, RQN ≥ 6.5, 28S:18S ≥ 1.0, >1 μg) was used to construct sequencing library. RNA purification, reverse transcription, library construction and sequencing were performed at Shanghai Majorbio Bio-pharm Biotechnology Co., Ltd. (Shanghai, China) according to the manufacturer’s instructions. The QJ RNA-seq transcriptome librariy was prepared following Illumina^®^ Stranded mRNA Prep, Ligation (San Diego, CA, USA) using 1 μg of total RNA. Shortly, mRNA was isolated according to polyA selection method by oligo(dT) beads and then fragmented by fragmentation buffer firstly. Secondly double-stranded cDNA was synthesized with random hexamer primers. Then the synthesized cDNA was subjected to end-repair, phosphorylation and adapter addition according to library construction protocol. Libraries were size selected for cDNA target fragments of 300–400 bp use magnetic beads followed by PCR amplified for 10–15 PCR cycles. After quantified by Qubit 4.0, the sequencing library was performed on NovaSeq X Plus platform (PE150) using NovaSeq Reagent Kit. The sequencing library was performed on DNBSEQ-T7 platform (PE150) using DNBSEQ-T7RS Reagent Kit (FCL PE150) version 3.0.

### 2.6. Quality Control and Read Mapping

The raw paired end reads were trimmed and quality controlled by fastp [29] with default parameters. Then clean reads were separately aligned to reference genome with orientation mode using HISAT2 (version 2.1.0) [30] software. The mapped reads of each sample were assembled by StringTie (version 2.2.3) [31] in a reference-based approach.

### 2.7. Differential Expression and Functional Enrichment Analyses

To identify DEGs between two different samples, the expression level of each transcript was calculated according to the transcripts per million reads (TPM) method. RSEM [32] was used to quantify gene abundances. Essentially, differential expression analysis was performed using the DESeq2 [33] or DEGseq [34]. DEGs with |log2FC| ≧ 1 and FDR < 0.05 (DESeq2) or FDR < 0.001 (DEGseq) were considered to be significantly DEGs. In addition, functional-enrichment analysis including GO and KEGG were performed to identify which DEGs were significantly enriched in GO terms and metabolic pathways at Bonferroni-corrected *p*-value < 0.05 compared with the whole-transcriptome background. GO functional enrichment and KEGG pathway analysis were carried out by Goatools (version 1.5.2) and Python SciPy (version 1.16.3) software, respectively.

### 2.8. Quantitative Reverse Transcription Polymerase Chain Reaction (qRT-PCR) Validation

To validate the transcriptome sequencing data, qRT-PCR was performed to assess the expression of 12 lignocellulolytic enzyme genes. Total RNA was extracted using the Juhemei RNAeasy kit (Juhemei, Beijing, China) according to the manufacturer’s instructions. The RNA was then reverse-transcribed into cDNA using the SweScript All-in-One RT SuperMix for qPCR kit (Vazyme Biotech Co., Ltd., Nanjing, China), and the resulting cDNA was diluted fivefold for use as the template. Subsequently, 2 μL of the diluted cDNA was added to a 20 μL reaction mixture prepared with the HiScript IV All-in-One Ultra RT SuperMix for qPCR kit (Vazyme, Nanjing, China), following a one-step PCR protocol. Quantitative PCR was carried out on a C1000 Touch™ CFX real-time PCR detection system (Bio-Rad Laboratories, Hercules, CA, USA). Target gene expression levels were normalized to that of the reference gene (α-tubulin), and relative expression levels were calculated using the 2^−ΔΔCt^ method. Primer sequences are provided in Supplementary Material Table S1.

### 2.9. Statistical Analysis

All experiments were performed in triplicate, and mean values were calculated. Statistical analyses were conducted using GraphPad Prism 10.6.0. The normality and homogeneity of variance of the data were first assessed. After confirming that the assumptions were met, one-way analysis of variance (ANOVA) was applied for multiple comparisons. Significant differences were further evaluated using Tukey’s test, and results were denoted by different letters. Differences were considered significant at *p* < 0.05.

## 3. Results

### 3.1. General Genome Features and Phylogenetic Comparison of I. lacteus QJ

**Figure 1:**
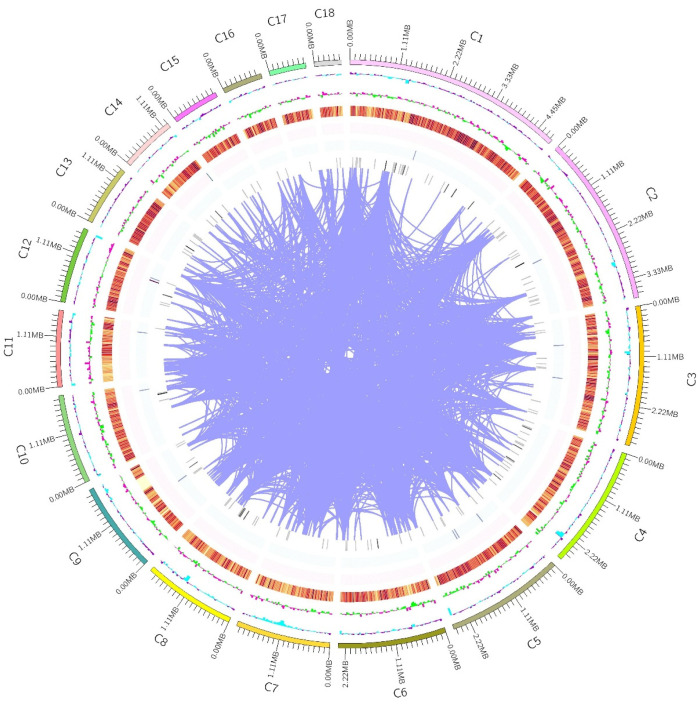
Overview of *I. lacteus* QJ genome. The outermost ring displays the genomic sequence coordinates. From the outer to the inner rings, the tracks represent the GC content, GC skew, gene density (including coding genes, rRNA, snRNA, and tRNA) and gene duplication, respectively.

Genomic DNA was extracted from the mycelia of *I. lacteus* QJ during the logarithmic growth phase. Whole-genome sequencing of *I. lacteus* QJ was performed using a combination of the Illumina and PacBio platforms. The assembled genome totaled 39,301,785 bp in length, comprising 45 scaffolds with a GC content of 50.67%, which is slightly lower than that of the previously sequenced reference strain *I. lacteus* F17 [35]. The largest scaffold was 4,836,332 bp, and the scaffold N50 value reached 2,087,568 bp. BUSCO assessment indicated a genome completeness of 99.7%, suggesting that the assembly is of high quality. Annotation statistics are summarized in Table 1. A total of 11,843 protein-coding genes were predicted, among which 6420 were annotated as hypothetical proteins, and the remaining genes (45.79%) were assigned putative functions. Compared to *I. lacteus* F17, the genome of QJ had a higher number of protein-coding genes and a greater proportion of coding sequence relative to the total genome length [35]. In addition, the *I. lacteus* QJ genome encoded 180 tRNA genes and 6 rRNA genes. The genomic circular map is presented in Figure 1.

**Figure 2:**
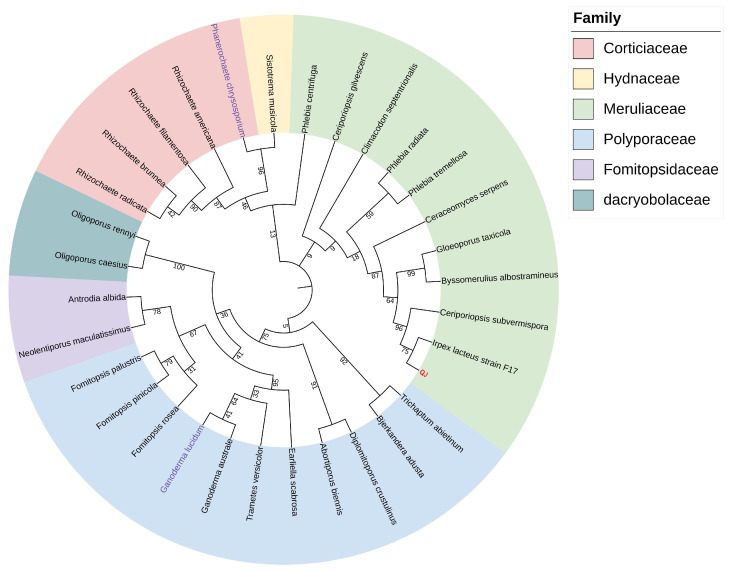
*I. lacteus* QJ’s phylogenetic tree within the family Polyporaceae. The strain marked in red is *I. lacteus* QJ. The strains marked in purple are two well-studied lignin-degrading fungi, *G. lucidum* and *P. chrysosporium*.

Phylogenetic reconstruction based on the conserved 18S rRNA gene confirmed *I. lacteus* QJ’s evolutionary divergence from certain strains within the family polyporaceae (Figure 2). The phylogenetic relationships between *I. lacteus* QJ and two major lignin-degrading fungi, *Ganoderma lucidum* and *P. chrysosporium*, were also investigated.

### 3.2. Gene Prediction and Classification

**Figure 3:**
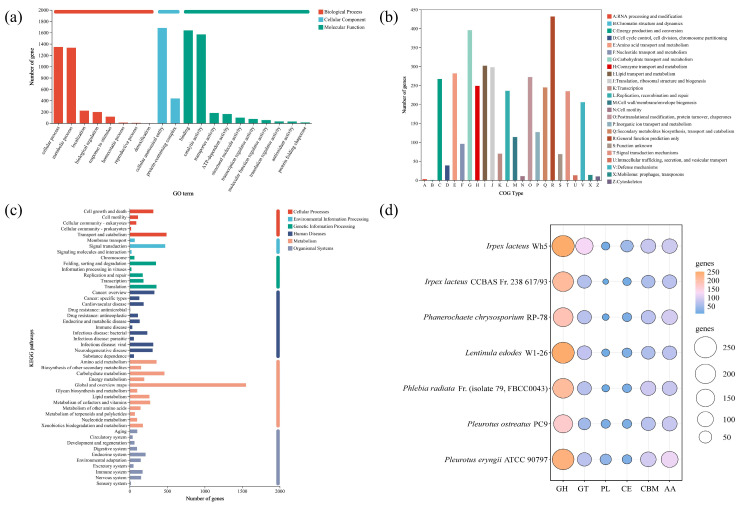
Functional annotation of *I. lacteus* QJ. (**a**) GO functional annotation of *I. lacteus* QJ. The *y*-axis indicates the gene number, and the *x*-axis denotes the three main GO categories including biological process, cellular component and molecular function, along with their respective subcategories. (**b**) COG functional annotation of *I. lacteus* QJ. The *y*-axis shows the number of matched coding genes, and the *x*-axis denotes the name of the function class. (**c**) KEGG functional annotation of *I. lacteus* QJ. The *y*-axis indicates the enriched pathway names, and the *x*-axis denotes the number of coding genes in the enriched pathways. (**d**) Carbohydrate-active enzymes functional classification and number of corresponding genes in *I. lacteus* QJ, *I. lacteus* CCBAS Fr. 238 617/93, *P. chrysosporium* RP-78, *L. edodes* W1-26, *P. radiata* Fr. (isolate 79, FBCC0043), *P. ostreatus* PC9, and *P. eryngii* ATCC 90797.

Functional annotation was performed on all 11,843 predicted protein-coding genes in *I. lacteus* QJ. Based on GO classification, a total of 3397 genes were assigned to the three major GO categories: biological process (BP), cellular component (CC) and molecular function (MF). As shown in Figure 3a, the top three enriched BP terms were cellular process (1347 genes), metabolic process (1333 genes), and localization (224 genes). GO analysis for CC revealed two major categories: cellular anatomical entity (1684 genes) and protein-containing complex (437 genes). The most abundant MF terms were binding (1641 genes), catalytic activity (1569 genes), and transporter activity (179 genes).

COG annotation assigned 3416 genes to 24 functional categories. The top five COG categories were general function prediction only (R, 10.8%), carbohydrate transport and metabolism (G, 9.9%), lipid transport and metabolism (I, 7.6%), translation, ribosomal structure and biogenesis (J, 7.5%), and amino acid transport and metabolism (E, 7.1%) (Figure 3b). The abundance of genes related to carbohydrate transport and metabolism suggests that *I. lacteus* QJ possesses strong capabilities for utilizing various carbohydrates and is likely to secrete a complex array of carbohydrate-degrading enzymes.

KEGG annotation successfully mapped 6200 genes, of which 3643 were assigned KEGG Orthology terms. Detailed KEGG pathway analysis (Figure 3c) revealed that these genes were enriched across six primary functional categories: metabolism, cellular processes, environmental information processing, genetic information processing, human diseases, and organismal systems. Among these genes, the metabolism category contained the largest number of genes (980). Within this category, 12 metabolic pathways were annotated, with 463 genes enriched in carbohydrate metabolism pathways. These included genes encoding endoglucanases, exoglucanases, and β-glucosidases [36]. Based on extensive literature and KEGG enrichment analysis, the carbohydrate metabolism pathways in *I. lacteus* QJ are closely associated with the production of lignocellulolytic enzymes [36].

### 3.3. Lignocellulolytic Enzymes of I. lacteus QJ

Based on the carbohydrate-active enzymes (CAZymes) database, 604 genes encoding CAZymes were annotated in *I. lacteus* QJ, including 251 glycoside hydrolases (GHs), 127 glycosyl transferases (GTs), 12 polysaccharide lyases (PLs), 46 carbohydrate esterases (CEs), 81 carbohydrate-binding modules (CBMs) and 87 auxiliary activity enzymes (AAs). Compared with *I. lacteus* CCBAS Fr. 238 617/93 [37], *P. chrysosporium* RP-78 [38], *Lentinula edodes* W1-26 [39], *Phlebia radiata* Fr. (isolate 79, FBCC0043) [40], *P. ostreatus* PC9 [41], and *Pleurotus eryngii* ATCC 90797 [42], QJ had a greater number of CAZyme genes, suggesting a stronger potential for lignocellulolytic enzyme secretion (Figure 3d, Supplementary Material Table S2). Comparison with *I. lacteus* CCBAS Fr. 238 617/93 further indicated that QJ had a relatively higher cellulase and hemicellulase secretion capacity among different *I. lacteus* strains. Figure 3d showed that QJ had more GH family genes than the six other white-rot fungi, consistent with reports that GH families contain many cellulase and hemicellulase [43].

Further analysis of CAZyme genes in QJ identified 13 cellulase genes and 25 hemicellulase genes, distributed among GH1, GH2, GH3, GH5, GH6, GH7, GH10, GH31, GH43, GH44, GH51, GH95 and CE1 families (Table 2). Compared to the other six white-rot fungi, QJ also encoded a larger number of GTs and CEs. CEs accelerate polysaccharide degradation by acting on ester bonds within carbohydrates, facilitating the entry of glycoside hydrolases. For example, acetylxylan esterases, key enzymes in lignocellulose degradation, are found in CE1, CE4, CE5, and CE16 families, all of which are represented in the QJ genome (Table 2). The identification of lignocellulolytic enzymes in *I. lacteus* QJ confirmed that all three key cellulase (6 endoglucanases, 4 exoglucanases, and 3 β-glucosidases) for cellulose degradation are encoded. Additionally, hemicellulase genes encoding endo-1,4-β-xylanase and β-xylosidase were identified. Genes encoding endo-1,4-β-mannosidase, endo-1,5-α-L-arabinosidase, and α-L-arabinofuranosidase, important for hemicellulose degradation, were also present (Table 2). The diverse repertoire of cellulases and hemicellulases highlighted the notable advantage of *I. lacteus* QJ in degrading complex polysaccharides. Lytic polysaccharide monooxygenases (LPMOs) were also closely associated with the degradation of cellulose and hemicellulose. According to NR and KEGG annotations, the QJ genome contained 16 LPMO genes.

As a typical white-rot fungus, *I. lacteus* is known for strong lignin degradation capability [43]. The main enzymes responsible for lignin degradation are lignin-modifying enzymes (LMEs), including laccases, MnP, LiP, VP, and DyP. Among *I. lacteus* QJ’s genome, 1 laccase gene, 9 MnP genes and 5 DyP genes were annotated. Besides LMEs, lignin degradation involves lignin-degrading auxiliary enzymes (LDAs). In the QJ genome, identified LDAs included glyoxal oxidase (GLX), cellobiose dehydrogenase (CDH), and pyranose 2-oxidase (P2O), which generate hydrogen peroxide, as well as cytochrome P450 monooxygenases (CYP450) that assist in degrading small aromatic compounds (Table 2). Previous studies have demonstrated that white-rot fungi can mineralize lignin into CO_2_, a process in which an organic polymer is progressively converted into inorganic end products. During this process, lignin is first depolymerized into low-molecular-weight, water-soluble intermediates and subsequently oxidized completely to CO_2_ and H_2_O through a non-specific extracellular oxidative system [16]. Our results similarly indicated that *I. lacteus* QJ possesses a highly efficient and complete ligninolytic enzyme system capable of oxidative cleavage of complex lignin polymers, sustained H_2_O_2_ supply to support peroxidase activity, and eventual mineralization of lignin-derived small molecules.

### 3.4. RNA-Sequencing-Based Transcriptome Analysis of the I. lacteus QJ

Studies have shown that the types of carbon sources affects the CAZyme activity of *I. lacteus* [24]. Transcriptomic analyses were performed under different carbon sources and at different time points to validate the genome annotation of *I. lacteus* QJ and analyze gene expression, especially that of lignocellulolytic enzymes. *I. lacteus* QJ was cultured in shake flasks using glucose or wheat straw as a carbon source with transcriptomes sampled and sequenced on days 4 and 8. Glucose was selected as a reference monosaccharide due to its ease of uptake and the relatively limited enzymatic machinery required for its metabolism, serving as a control to assess constitutive CAZyme gene expression. Wheat straw, composed mainly of lignocellulose, represents a complex heterogeneous substrate that requires diverse CAZymes for degradation. Importantly, wheat straw also holds significant potential as a feedstock for biorefinery applications.

**Figure 4:**
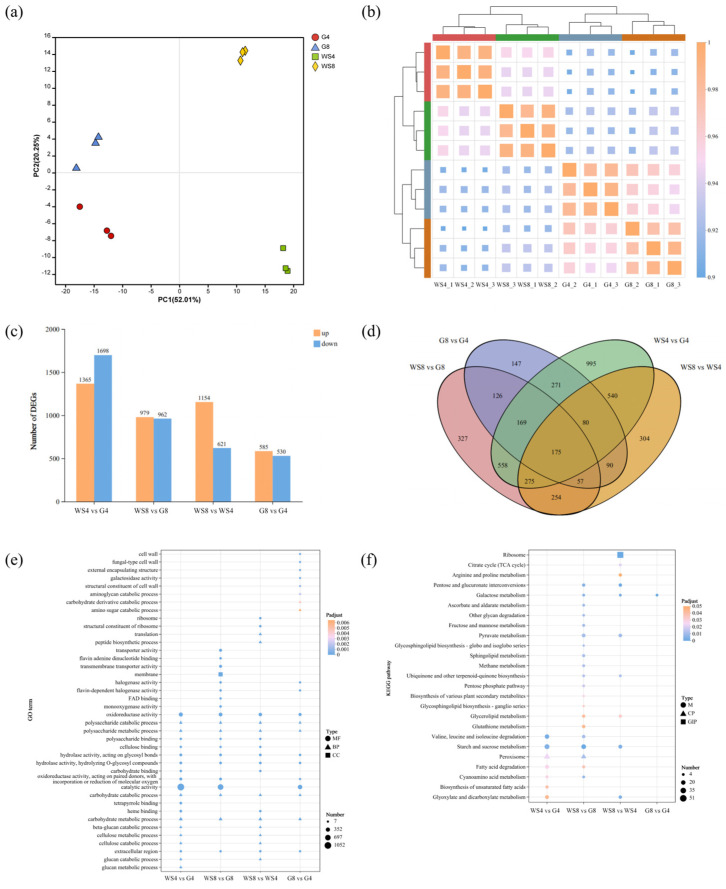
Transcriptomic data mining of *I. lacteus* QJ cultured with glucose or wheat straw for 4 or 8 days. (**a**) PCA clustering of the four sample groups. (**b**) Correlation analysis of expression levels among the four sample groups. (**c**) Distribution of DEGs across the four comparisons. (**d**) Venn diagram showing shared DEGs among the four comparisons. (**e**) GO term enrichment comparison of DEGs in four comparisons. (**f**) KEGG pathway enrichment comparison of DEGs in four comparisons.

Samples were divided into four groups according to carbon source and sampling time: G4 (glucose, day 4), WS4 (wheat straw, day 4), G8 (glucose, day 8), and WS8 (wheat straw, day 8). Illumina sequencing generated a total of 652.49 million raw reads. After filtering and quality control, 645.81 million clean reads remained, with Q30 base quality scores exceeding 94.84% (Supplementary Material Table S3). Principal component analysis (PCA) of 12 samples revealed that PC1 and PC2 accounted for 52.01% and 20.25% of variance, respectively (Figure 4a). The smallest difference was observed between G4 and G8, whereas differences between WS4 and G4 and between WS8 and G8 were substantial. Correlation analysis showed stronger relationships between groups with the same carbon source than the groups with the same sampling time (Figure 4b).

To further explore DEGs influenced by carbon source and time, four pairwise comparisons were conducted: WS4 vs. G4, WS8 vs. G8, WS8 vs. WS4, and G8 vs. G4. The greatest number of DEGs (3063) was identified between WS4 and G4, with 1365 genes upregulated and 1698 downregulated. The fewest DEGs (1115) were found between G8 and G4, including 585 upregulated and 530 downregulated genes. In the other two comparisons, WS8 vs. G8 showed 979 upregulated and 962 downregulated genes, while WS8 vs. WS4 had 1154 upregulated and 621 downregulated genes (Figure 4c). A Venn diagram revealed 175 overlapping DEGs across all four comparisons (Figure 4d). The largest overlap (1177 DEGs) occurred between WS4 vs. G4 and WS8 vs. G8, which suggested that the DEGs affected by carbon source are relatively consistent. The smallest overlap (402 DEGs) was between WS8 vs. WS4 and G8 vs. G4. Collectively, these analyses indicated that growth conditions strongly influence the transcriptome, with shifts in carbon source inducing or repressing a larger set of genes. Moreover, the effect of carbon source type on gene expression in *I. lacteus* QJ is sustained over time, impacting a relatively stable set of DEGs.

### 3.5. KEGG Pathway and GO Enrichment Analyses of the DEGs

To analyze the expression patterns of DEGs, GO and KEGG enrichment analyses were performed for the four comparison groups (WS4 vs. G4, WS8 vs. G8, WS8 vs. WS4, and G8 vs. G4), and their enrichment profiles were compared. In the WS4 vs. G4 comparison, the major enriched GO terms included catalytic activity (1052), membrane (670), and oxidoreductase activity (316), while the key KEGG pathways were starch and sucrose metabolism (26), peroxisome (26), and valine, leucine and isoleucine degradation (21). For WS8 vs. G8, prominent GO terms were molecular function (924), catalytic activity (696), and membrane (462), with starch and sucrose metabolism (24), peroxisome (17), and pyruvate metabolism (15) enriched in KEGG. In WS8 vs. WS4, enriched GO terms included catalytic activity (580), membrane (367), and oxidoreductase activity (206); the enriched KEGG pathways were ribosome (51), starch and sucrose metabolism (16), and pyruvate metabolism (14). In G8 vs. G4, the major GO terms were catalytic activity (401), hydrolase activity (175), and oxidoreductase activity (138), and the KEGG pathways included starch and sucrose metabolism (11), amino sugar and nucleotide sugar metabolism (10), and glycerophospholipid metabolism (9) (Supplementary Materials Tables S4 and S5). These results indicated that across all comparisons, DEGs related to carbohydrate metabolism and oxidoreductase functions were highly enriched, indicating that sample differences are closely related to carbon metabolism. Notably, more DEGs associated with lignocellulose degradation were enriched in groups cultured with different carbon sources but sampled at the same time.

To further examine differences in DEG enrichment among the comparison groups, a multi-group GO and KEGG enrichment comparison was conducted. Analysis of GO enrichment revealed that, compared to the other three groups, the G8 vs. G4 comparison showed many DEGs involved in the synthesis of fungal cell walls, such as fungal-type cell wall, external encapsulating structure, and structural constituent of cell wall (Figure 4e). The result suggested that when glucose is used as the carbon source, cultivation time affects the cell life cycle, while transcription levels of lignocellulolytic enzyme genes remain relatively stable, resulting in fewer DEGs. In contrast, the WS8 vs. WS4 comparison showed enrichment of many DEGs in GO terms related to lignocellulose degradation, such as oxidoreductase activity, carbohydrate catabolic process, and carbohydrate metabolic process (Figure 4e). This profile was significantly different from that of the G8 vs. G4 comparison. Compared to the other three comparisons, DEGs in WS8 vs. WS4 were also enriched in GO terms linked to secretion and extracellular protein transport. It likely reflected that the expression of various degradative enzymes changed over time when wheat straw was used as the carbon source, and secreted protein levels were significantly impacted. Comparing GO enrichment between WS8 vs. WS4 and G8 vs. G4 revealed that wheat straw exerts a stronger induction effect on lignocellulolytic enzymes than glucose (Figure 4e). Correspondingly, fewer DEGs related to cell growth and life cycle were enriched in *I. lacteus* QJ when cultured on wheat straw, implying that wheat straw may prolong the cell life cycle or affect cell growth and proliferation. Consistent with this, during shake-flask fermentation, biomass in WS groups was noticeably lower than that in G groups (*p* < 0.05, Supplementary Material Figure S1).

Compared to the G8 vs. G4 and WS8 vs. WS4, the DEGs in WS4 vs. G4 and WS8 vs. G8 primarily resulted from the difference in carbon source, with most DEGs in these comparisons enriched in GO terms related to lignocellulose degradation (Figure 4e). This indicated a significant difference in the expression patterns of lignocellulolytic enzyme genes in *I. lacteus* QJ when cultured on wheat straw versus glucose. Interestingly, relative to WS8 vs. G8, WS4 vs. G4 showed more DEGs annotated to GO terms related to cellulose and hemicellulose degradation, including carbohydrate binding, β-glucan catabolic process, cellulose metabolic process, cellulose catabolic process, glucan catabolic process and glucan metabolic process (Figure 4e). This suggested that on day 4, wheat straw primarily induces expression of cellulase and hemicellulose genes, with minimal induction of ligninolytic enzyme genes. The DEG distribution in WS8 vs. G8 followed a complementary pattern, with more DEGs related to lignin degradation were enriched, such as flavin adenine dinucleotide binding, FAD binding, and monooxygenase activity (Figure 4e). This indicated that carbon source-induced differences in ligninolytic enzyme gene expression became more pronounced on day 8, while differences in cellulase and hemicellulose genes expression decreased. Overall, wheat straw strongly regulated lignocellulolytic enzyme gene expression in a time-dependent manner compared to glucose. Cellulase and hemicellulase genes were mainly induced on day 4, whereas ligninolytic enzyme genes were induced on day 8. These findings suggested that differential expression of genes involved in cellulose and hemicellulose degradation (or their intermediate or final products) may be prerequisites for subsequent lignin degradation.

Additionally, KEGG enrichment analyses further supported these observations. DEGs in WS4 vs. G4 and WS8 vs. G8 were significantly enriched in the starch and sucrose metabolism and peroxisome pathways (Figure 4f). Specifically, WS4 vs. G4 showed 26 DEGs enriched in each pathway, while WS8 vs. G8 had 24 and 17 DEGs, respectively, enriched in starch and sucrose metabolism and peroxisome pathways. This confirmed that wheat straw as a carbon source exerts significant regulatory effects on lignocellulolytic enzyme gene expression.

### 3.6. Expression Patterns of Lignocellulolytic Enzyme Genes Under Different Cultivation Conditions

Studies on the enrichment of DEGs offer only a broad overview of gene expression changes and their general induction trends in response to carbon source and time. To explore more precisely how lignocellulolytic enzyme gene expression is influenced by carbon source and cultivation duration, we performed clustering analysis on four enzyme gene categories (cellulases, hemicellulases, LPMOs, and ligninolytic enzymes) previously identified from the genome. This allowed us to compare expression patterns across different experimental groups.

**Figure 5:**
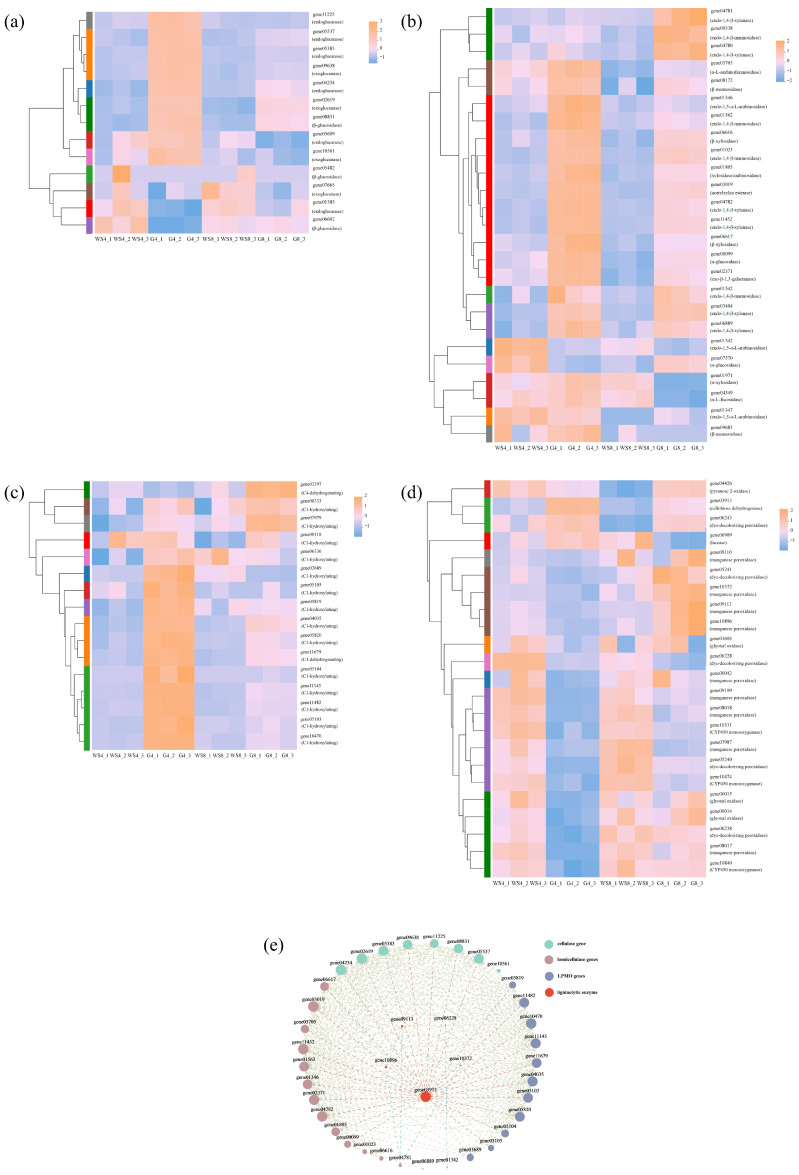
Expression patterns of potential lignocellulolytic enzymes in *I. lacteus* QJ. (**a**) Transcriptomic gene expression of cellulase genes. (**b**) Transcriptomic gene expression of hemicellulase genes. (**c**) Transcriptomic gene expression of LPMO genes. (**d**) Transcriptomic gene expression of ligninolytic enzyme genes. (**e**) Lignocellulolytic enzyme genes with potential expression relevance. Two connected points exhibited expression correlation. The yellow lines represented expression correlations among polysaccharide-degrading enzyme genes, the red lines represented expression correlations between CDH gene and other genes, and the blue lines represented expression correlations between ligninolytic enzyme genes (other than CDH gene) and other genes. the larger the corresponding gene point, the greater the number of genes exhibiting expression correlation.

The expression patterns of cellulase genes are shown in Figure 5a. Among the 13 identified cellulase genes, five endoglucanase genes (gene04254, gene05383, gene05537, gene05609, and gene11225), three exoglucanase genes (gene02619, gene09638, and gene10561), and one β-glucosidase gene (gene08831) exhibited the highest expression in the WS4 group, followed by WS8, with much lower expression levels in the G4 and G8 groups. Expression patterns in G4 and G8 were also relatively similar. Among the 25 hemicellulase genes, 18 genes displayed strong expression in the WS4 group. These included three endo-1,4-β-xylanase genes (gene04782, gene11452, and gene06889), two β-xylosidase genes (gene06616 and gene06617), three endo-1,4-β-mannosidase genes (gene01023, gene01542, and gene01562), two β-mannosidase genes (gene08172 and gene09683), one endo-1,5-α-L-arabinosidase gene (gene01346), one α-L-arabinofuranosidase gene (gene05705), and six other hemicellulase genes (gene01805, gene02371, gene00099, gene01971, gene03019, and gene04349) (Figure 5b). Among 16 LPMO genes, 13 genes (gene00110, gene03689, gene04035, gene05103, gene05104, gene05105, gene05819, gene05820, gene06336, gene10470, gene11145, gene11482, and gene11679) were highly expressed in the WS4 group (Figure 5c). These results indicated that most polysaccharide-degrading enzyme genes, including those encoding cellulases, hemicellulases, and LPMOs, share a similar expression pattern, characterized by peak expression in WS4. Overall, polysaccharide-degrading enzyme gene expression levels in WS groups were consistently higher than those in G groups, and expression in G4 and G8 was low or nearly undetectable. Consistent with this performance, the WS4 vs. G4 group comparison exhibited a higher prevalence of DEGs enriched in modules associated with polysaccharide degradation in the GO enrichment profiles than other three pairwise comparisons (Figure 4e). This consistency further supports a time-dependent induction of polysaccharide-degrading enzyme gene expression by carbon source. These findings suggested that wheat straw as a carbon source significantly induces polysaccharide-degrading enzyme gene expression, particularly on day 4, with lower expression observed on day 8.

Interestingly, among genes involved in lignin degradation, only one CDH gene (gene03913) and one dye-decolorizing peroxidase gene (gene06243) exhibited expression patterns similar to those of polysaccharide-degrading enzyme genes. In contrast, most ligninolytic enzyme genes were expressed at higher levels in the G groups than in the WS groups (Figure 5d). Furthermore, when comparing WS8 to WS4, 12 of the 14 LME genes showed higher expression in WS8. Among them, four MnP genes (gene09110, gene09113, gene10372, and gene10896) and one DyP gene (gene05241) were more highly expressed in WS8 than in WS4, G4, or G8. In general, ligninolytic enzyme genes responded differently to carbon source compared to polysaccharide-degrading enzyme genes, and their expression was greater on day 8 than on day 4 when wheat straw was used. This suggested a delayed peak in ligninolytic enzyme gene expression relative to other lignocellulolytic enzymes. These findings implied that ligninolytic enzyme gene regulation differed from polysaccharide-degrading enzyme gene regulation under wheat straw.

To explore potential interactions between polysaccharide-degrading enzyme and ligninolytic enzyme gene expression, Pearson analysis (|r| ≥ 0.95, *p* < 0.05) was performed on all annotated lignocellulolytic enzyme genes in Table 2. A total of 39 lignocellulolytic enzyme genes exhibited significant expression correlations with other genes. Among ligninolytic enzyme genes, the CDH gene (gene03913) was co-expressed with 26 polysaccharide-degrading enzyme genes, including six cellulase genes, ten hemicellulase genes, and ten LPMO genes (Figure 5e). This confirmed that wheat straw not only induces polysaccharide-degrading enzyme gene expression but also promotes CDH gene expression. As a member of the LDA family, CDH is known to generate H_2_O_2_, which is essential for the catalytic activity of peroxidases in the LME family. Therefore, the high expression of CDH on day 4 may contribute to hydrogen peroxide production, which in turn facilitates the elevated expression of LMEs observed on day 8.

### 3.7. Validation of Transcriptomic Data by qRT–PCR

**Figure 6:**
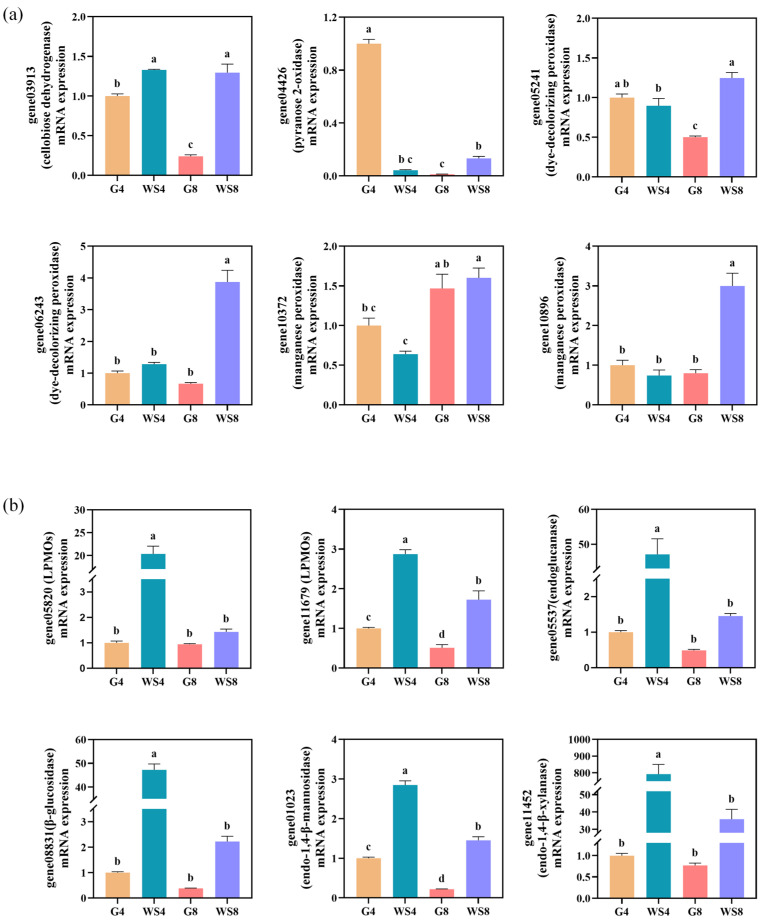
qRT-PCR validation of mRNA expression levels of 12 selected genes in the *I. lacteus* QJ transcriptome. (**a**) Six ligninolytic enzyme genes. (**b**) Six polysaccharide-degrading enzyme genes. ^a–d^ represent different significant differences (*p* < 0.05).

To validate the transcriptomic results, qRT-PCR analysis was performed. Based on gene expression patterns, a subset of DEGs involved in lignin, cellulose, and hemicellulose metabolism (Table 1) was selected for analysis. Six ligninolytic enzyme genes (gene03913, gene04426, gene05241, gene06243, gene10372, and gene10896) were examined (Figure 6a). The expression profiles of these genes were similar to the transcriptome data (Figure 5d). In particular, four LME-encoding genes (gene05241, gene06243, gene10372, and gene10896) were significantly upregulated in the WS8 group (*p* < 0.05). In addition, the expression patterns of six polysaccharide-degrading enzyme genes (gene05820, gene11679, gene05537, gene08831, gene01023, and gene11452) were verified (Figure 6b). All six genes were significantly upregulated in the WS4 group (*p* < 0.05), consistent with the transcriptomic expression profiles (Figure 5a–c).

## 4. Discussion

*I. lacteus* is an efficient lignocellulose degrader in nature that predominantly colonize decaying wood and belongs to the white-rot fungi [43]. White-rot fungi are a relatively rare group of filamentous fungi capable of completely degrading all components of the plant cell wall [44]. Nearly all of the white-rot fungi are Basidiomycetes, with the most extensively studied species belonging to the order Polyporales, while a few are classified under Agaricales, Auriculariales, Hymenochaetales, and Russulales [45]. The *I. lacteus* strain QJ is taxonomically assigned to Polyporales [46]. Phylogenetic analysis based on 18S rRNA sequences revealed that QJ was closely related to *I. lacteus* F17 (Figure 2). The phylogenetic tree also compared QJ with other Polyporales fungi, notably *P. chrysosporium* and *G. lucidum*. *P. chrysosporium* is a model species for white-rot fungi research, with nearly 2000 related publications (1945 articles retrieved from PubMed by species name), and is frequently used as a reference in comparative studies of other species [44]. *G. lucidum*, an important medicinal mushroom, has been shown to efficiently utilize lignocellulose for growth, enabling its cultivation on high-fiber waste substrates to reduce costs and recycle waste materials [47]. Comparative 18S rRNA analysis demonstrated that *I. lacteus* QJ is more closely related to *P. chrysosporium* than to *G. lucidum* (Figure 2).

Among numerous white-rot fungi, *I. lacteus* exhibits not only excellent lignin-degrading capacity but also strong cellulolytic and hemicellulolytic activities [18,24]. To investigate the genes involved in lignocellulose degradation and their regulatory mechanisms, we sequenced and analyzed the genome of *I. lacteus* QJ, identifying genes encoding enzymes in lignocellulose degradation. Genome analysis revealed that QJ has a genome size of 39.30 Mb (Table 1), smaller than that of *I. lacteus* F17 (44.36 Mb) and *I. lacteus* CD2 (43.16 Mb) [43]. Notably, QJ encoded the highest number of glycoside hydrolase (GH) family genes (Supplementary Material Table S2), which are closely associated with cellulose and hemicellulose degradation. Specifically, F17 encoded 161 GHs and CD2 encoded 182 GHs, whereas QJ possessed even more GH-encoding genes, suggesting a relatively higher capacity for cellulose and hemicellulose degradation compared to F17 and CD2 strains.

The ability of QJ to express a large repertoire of GHs enzymes presents both advantages and challenges in industrial applications. On the one hand, increased secretion of cellulases and hemicellulases promotes fungal growth and releases more soluble sugars from cellulose and hemicellulose, thereby enhancing the efficiency of lignocellulose pretreatment [44]. However, on the other hand, excessive expression of GHs may lead to the preferential utilization of cellulose and hemicellulose as carbon sources, reducing the selectivity of lignin degradation by white-rot fungi, and diverting carbohydrates toward CO_2_ production and loss, ultimately decreasing the nutritional and energy value of the substrate [16].

To better understand the temporal sequence and mutual regulation of cellulases, hemicellulases, and ligninolytic enzymes in QJ, and to control carbon flux during lignocellulose degradation, further transcriptomic analysis of QJ was conducted. The sequenced and annotated genome of *I. lacteus* QJ provided an excellent platform for subsequent transcriptomic analyses. In this work, the high-fiber carbon source used for fungal cultivation consisted of wheat straw segments approximately 10 mm in length, which were cleaned to remove most surface dust and had the uniform quality of the raw material. Submerged fermentation was employed instead of solid-state fermentation to ensure thorough contact between secreted enzymes and the substrate, which could increase sample yield and shorten the fermentation cycle. Wheat straw was chosen as a representative lignocellulosic substrate because previous studies have shown that *I. lacteus* possesses a strong capacity for its degradation [24,44]. Moreover, QJ was originally isolated from wheat straw, suggesting that this substrate can serve as a suitable carbon source for its normal growth.

When wheat straw was used as the carbon source, the transcriptomic differences between day 8 and day 4 were greater than those observed with glucose (1775 DEGs for WS8 vs. WS4, compared with 1115 DEGs for G8 vs. G4). This suggested that the carbon source had a significant influence on the number of DEGs over cultivation time. The differences between the WS8 and WS4 groups were not solely attributable to distinct physiological stages of the fungus, but also to substrate decomposition and temporal changes in the sugar profile [48]. Enrichment analysis of DEGs identified between different carbon sources further revealed that the DEGs affected by carbon source were primarily associated with cellulose and hemicellulose degradation on day 4, while a subset of DEGs was associated with lignin degradation on day 8. Enzyme activity assays for another *I. lacteus* strain, CD2, similarly showed that the peak activities of cellulases and hemicellulases all occurred on days 6–9, while the peak activity of ligninolytic enzymes occurred at a different time point [43]. These findings indicate that, when lignocellulosic biomass is used as a carbon source, the expression and regulation patterns of GH genes are relatively consistent, whereas ligninolytic enzymes follow a distinct regulatory pattern. The phenomenon of asynchronous expression timing between polysaccharide-degrading enzyme genes and ligninolytic enzyme genes has been observed in the transcriptome of *Trichoderma asperellum* CGMCC 41476, an ascomycete capable of lignocellulose degradation. When cultured on NaOH-pretreated wheat straw under solid-state fermentation, the peak expression of cellulases and hemicellulases occurred on days 5 and 10, respectively, while the peak expression of manganese peroxidase was observed on day 30 [49].

Regulation of cellulase and hemicellulase expression by carbon source is primarily mediated through carbon catabolite repression (CCR), in which simple carbon sources such as glucose inhibit the expression of these enzyme genes [50]. Comparative analysis of DEGs between different carbon source groups in the transcriptome of *I. lacteus* QJ revealed that ligninolytic enzyme genes are not subject to glucose-induced CCR. Indeed, proteomic analysis of *P. ostreatus* under different carbon sources has shown that simple sugars such as glucose do not affect ligninolytic enzyme secretion, although certain oxidoreductases, such as CDH, are influenced by CCR [51]. This may relate to their auxiliary role in cellulose degradation.

In QJ, differential expression analysis of key enzyme genes across treatment groups showed that the CDH gene (gene03913), which is associated with lignin degradation, displayed an expression pattern similar to those of cellulase, hemicellulase, and LPMOs genes, but distinct from other ligninolytic enzyme genes. The CDH gene exhibited expression correlations with those of 26 polysaccharide-degrading enzyme genes (Figure 5e). CDH plays an important role in polysaccharide degradation, acting as an electron donor for LPMOs. Previous studies have demonstrated that CDH generates H_2_O_2_ through redox interactions with LPMOs, and then LPMOs use H_2_O_2_ to cleave polysaccharide substrates [52,53]. Furthermore, CDH-derived H_2_O_2_ is essential for LMEs in lignin degradation. Supplementation of culture media with H_2_O_2_ has been shown to enhance the activities of LiP and MnP, thereby improving LME function [54]. Based on expression patterns of related enzyme genes, we infer that in the later stages of QJ cultivation, the interaction between CDH and LPMOs leads to the accumulation of high levels of H_2_O_2_, which subsequently induces MnP expression, resulting in a delayed upregulation of ligninolytic enzyme genes compared with CDH gene and other polysaccharide-degrading enzyme genes.

**Figure 7:**
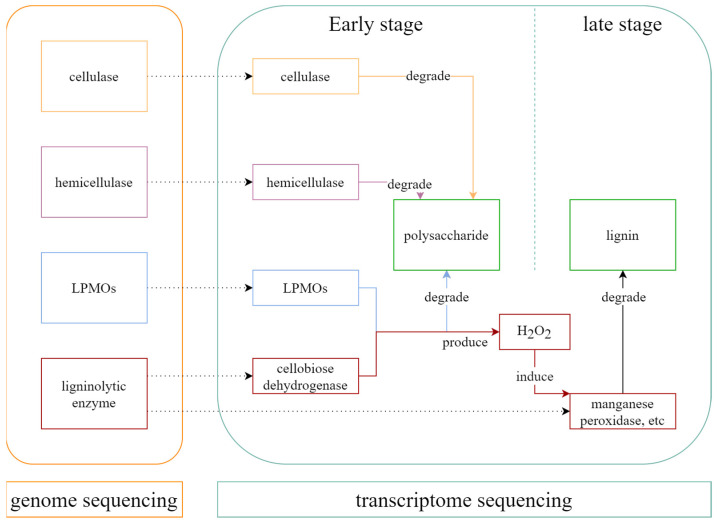
Gene expression profiles of lignocellulolytic enzyme genes in *I. lacteus* QJ.

In summary, based on genome and transcriptome analyses, we have gained further insight into lignocellulose degradation by *I. lacteus* QJ under submerged fermentation. In the early stage (approximately day 4), QJ predominantly expresses cellulase and hemicellulase genes. These enzymes are rapidly produced and accumulated, initiating the attack on the lignocellulose network, degrading cellulose and hemicellulose components, and disrupting the ordered structure of lignocellulose. At the same time, high expression of CDH and LPMO genes generates H_2_O_2_, contributing to cellulose and hemicellulose degradation. In the later stage (approximately day 8), the accumulated H_2_O_2_ induces MnP expression, enabling the direct oxidation of phenolic lignin compounds and causing more extensive structural damage to lignocellulose. A schematic overview of the lignocellulose degradation mechanism by *I. lacteus* QJ is presented in Figure 7. Based on existing research and our experiments, we infer that CDH plays a bridging role in lignocellulose degradation through interaction with LPMOs [55,56]. CDH not only promotes the degradation of cellulose via redox reactions but also enhances the function of ligninolytic enzymes by producing H_2_O_2_ [52,53,54]. These results provide a theoretical basis for production, showing how enzyme regulation during lignocellulose degradation by *I. lacteus*. Based on this insight, the addition of a small amount of H_2_O_2_ may help promote the secretion of ligninolytic enzymes and enhance the degradation of lignocellulosic substrates. However, the mechanisms of H_2_O_2_ generation and its regulation of ligninolytic enzyme gene expression are complex and require further investigation. Given that high concentrations of H_2_O_2_ can be detrimental to both enzymes and fungal cells, strategies aimed at enhancing lignin degradation by increasing H_2_O_2_ levels should be approached with caution [25].

## 5. Conclusions

In summary, this study sequenced the genome of *I. lacteus* QJ and constructed a phylogenetic tree based on the 18S rRNA gene. Genome analysis revealed that *I. lacteus* QJ possesses a rich repertoire of CAZyme genes, among which 77 were annotated as being involved in lignocellulose degradation. To further investigate the regulatory mechanisms of these enzymes during lignocellulose degradation, *I. lacteus* QJ was cultured with different carbon sources for varying durations, followed by transcriptome sequencing. The results showed that under different carbon source conditions, the enriched DEGs were closely associated with lignocellulose degradation processes. Moreover, the enrichment patterns of DEGs differed across the four comparison groups, indicating that lignocellulose degradation exhibits temporal variation. Expression profiling of the annotated enzyme genes showed that when wheat straw served as the carbon source, polysaccharide-degrading enzyme genes together with the CDH gene were activated earlier, likely because they were jointly induced by the carbon source. In contrast, ligninolytic enzyme genes exhibited a delayed expression pattern, which may be attributable to their regulation by CDH-derived H_2_O_2_, suggesting that these genes are potentially induced by H_2_O_2_. Although the key advantages that may distinguish *I. lacteus* from other white-rot fungi in lignocellulose degradation remain unclear, our findings expand current knowledge of its enzyme genes and their expression patterns. These findings in this study enhance our understanding of the regulatory mechanisms employed by *I. lacteus* QJ during lignocellulose degradation and provide a valuable reference for optimizing degradation conditions in fermentation-based production.

## Figures and Tables

**Table 1:** Comparison of genome characteristics between *I. lacteus* QJ and the model strain *I. lacteus* F17.

Sample Name	*Irpex lacteus* QJ	*Irpex lacteus* F17
Total Scaffold No.	45	-
Total Bases in Scaffold (bp)	39,301,785	44,362,654
Scaffold N50 (bp)	2,087,568	-
Scaffold N90 (bp)	885,300	-
G + C (%)	50.67	49.64
Gene No.	11,843	10,661
Gene Total Length (bp)	26,809,457	15,030,327
Gene/Genome (%)	68.21	33.88
BUSCO Complete (%)	98.3	-
BUSCO Complete Duplicated (%)	0.3	-
BUSCO Fragmented (%)	0.5	-
BUSCO Missing (%)	1.2	-

Note: ‘-’ indicates that the relevant data were not reported in the references.

**Table 2:** List of potential lignocellulolytic enzymes found in the genome of *I. lacteus* QJ.

Category	Enzyme Class	Gene No.	CAZyme Family	Gene_ID
Cellulase	endoglucanase	6	GH5	gene01385
				gene05383
				gene05537
				gene05609
				gene11225
			GH44	gene04254
	exoglucanase	4	GH6	gene02619
			GH7	gene07665
				gene09638
				gene10561
	β-glucosidase	3	GH1	gene05482
				gene08831
			GH3	gene06602
Hemicellulase	endo-1,4-β-xylanase	6	GH10	gene03404
				gene04780
				gene04781
				gene04782
				gene11452
			GH43	gene06889
	β-xylosidase	2	GH5	gene06616
				gene06617
	endo-1,4-β-mannosidase	4	GH5	gene00338
				gene01023
				gene01542
				gene01562
	β-mannosidase	2	GH2	gene08172
				gene09683
	endo-1,5-α-L-arabinosidase	3	GH43	gene01342
				gene01346
				gene01347
	α-L-arabinofuranosidase	1	GH51	gene05705
	xylosidase/arabinosidase	1	GH43	gene01805
	exo-β-1,3-galactanase	1	GH43	gene02371
	α-glucosidase	2	GH31	gene00099
				gene07570
	α-xylosidase	1	GH31	gene01971
	acetylxylan esterase	1	CE1	gene03019
	α-L-fucosidase	1	GH95	gene04349
LPMOs	lytic cellulose monooxygenase	16	AA9	gene00110
				gene03689
				gene04035
				gene05103
				gene05104
				gene05105
				gene05819
				gene05820
				gene06333
				gene06336
				gene07979
				gene10470
				gene11145
				gene11482
				gene03397
				gene11679
ligninolytic enzyme	laccase	1	AA1	gene06909
	manganese peroxidase	9	AA2	gene00042
				gene07987
				gene08017
				gene08018
				gene09109
				gene09110
				gene09113
				gene10372
				gene10896
	glyoxal oxidase	3	AA5	gene00014
				gene00015
				gene01604
	dye-decolorizing peroxidase	5		gene05240
				gene05241
				gene06228
				gene06238
				gene06243
	CYP450 monooxygenase	3		gene10474
				gene10531
				gene10840
	cellobiose dehydrogenase	1	AA8	gene03913
	pyranose 2-oxidase	1	AA3	gene04426

## Data Availability

The data presented in this study are openly available in the Genome Sequence Archive (Genomics, Proteomics & Bioinformatics 2025) in National Genomics Data Center (Nucleic Acids Res 2025), China National Center for Bioinformation/Beijing Institute of Genomics, Chinese Academy of Sciences at https://ngdc.cncb.ac.cn/gsa (accessed on 19 October 2025), reference number GSA: CRA031489 and CRA031472 [57,58].

## Supplementary Materials

The following supporting information can be downloaded at: https://www.mdpi.com/article/10.3390/jof11120882/s1, Table S1: Candidate gene primer sequences; Table S2: Comparison of the number of CAZy genes encoded in the genomes of *Irpex lacteus* QJ and six other filamentous fungi; Table S3: Transcriptome sequencing data for four groups; Table S4: GO enrichment analysis; Table S5: KEGG enrichment analysis; Figure S1: Dry weight of *Irpex lacteus* QJ cultured in 100 mL MA medium on day 4.
