# Supplementary figures and images for: Genomic and Transcriptomic Insights into Carbon-Source and Temporal Induction of a Diverse Set of Lignocellulolytic Enzymes in Irpex lacteus QJ
Source: J Fungi (Basel). 2025 Dec 13;11(12):882. doi: 10.3390/jof11120882 (PMC12733758; doi:10.3390/jof11120882)

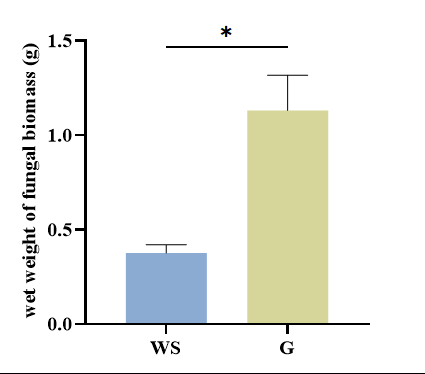

Supplement: Supplementary file 1 [file jof-11-00882-s001.zip › Supplementary Material Figure S1. Dry weight of Irpex lacteus QJ cultured in 100 ml MA medium on day 4.png]
